# Non-Temperature Induced Effects of Magnetized Iron Oxide Nanoparticles in Alternating Magnetic Field in Cancer Cells

**DOI:** 10.1371/journal.pone.0156294

**Published:** 2016-05-31

**Authors:** Sudath Hapuarachchige, Yoshinori Kato, Ethel J. Ngen, Barbara Smith, Michael Delannoy, Dmitri Artemov

**Affiliations:** 1 Division of Cancer Imaging Research, The Russell H. Morgan Department of Radiology and Radiological Science, The Johns Hopkins University School of Medicine, Baltimore, Maryland, 21205, United States of America; 2 Department of Oncology, The Sidney Kimmel Comprehensive Cancer Center, The Johns Hopkins University School of Medicine, Baltimore, Maryland, 21287, United States of America; 3 Cell Biology Imaging Facility, The Johns Hopkins University School of Medicine, Baltimore, Maryland, 21205, United States of America; Brandeis University, UNITED STATES

## Abstract

This paper reports the damaging effects of magnetic iron-oxide nanoparticles (MNP) on magnetically labeled cancer cells when subjected to oscillating gradients in a strong external magnetic field. Human breast cancer MDA-MB-231 cells were labeled with MNP, placed in the high magnetic field, and subjected to oscillating gradients generated by an imaging gradient system of a 9.4T preclinical MRI system. Changes in cell morphology and a decrease in cell viability were detected in cells treated with oscillating gradients. The cytotoxicity was determined qualitatively and quantitatively by microscopic imaging and cell viability assays. An approximately 26.6% reduction in cell viability was detected in magnetically labeled cells subjected to the combined effect of a static magnetic field and oscillating gradients. No reduction in cell viability was observed in unlabeled cells subjected to gradients, or in MNP-labeled cells in the static magnetic field. As no increase in local temperature was observed, the cell damage was not a result of hyperthermia. Currently, we consider the coherent motion of internalized and aggregated nanoparticles that produce mechanical moments as a potential mechanism of cell destruction. The formation and dynamics of the intracellular aggregates of nanoparticles were visualized by optical and transmission electron microscopy (TEM). The images revealed a rapid formation of elongated MNP aggregates in the cells, which were aligned with the external magnetic field. This strategy provides a new way to eradicate a specific population of MNP-labeled cells, potentially with magnetic resonance imaging guidance using standard MRI equipment, with minimal side effects for the host.

## Introduction

Applications for magnetic nanoparticles (MNP), such as superparamagnetic iron oxide nanoparticles (SPION), in biomedicine are continuously expanding due to their unique properties, which include: biocompatibility and magnetic interaction with external magnetic fields that can generate imaging contrast in magnetic resonance imaging (MRI) [1,2,3], as well as thermal [4] and mechanical effects [5,6]. Mammalian cells can be efficiently loaded with MNP using various labeling protocols [3,7,8]. The MRI contrast generated by MNP has been successfully used for MR tracking of transplanted cells in preclinical models [9,10,11] and clinical settings [12]. Typical iron concentrations in the range of 5–10 pg iron/cell, used for *in vivo* MRI, do not seem to result in cytotoxicity or impeded differentiation of pluripotent stem cells [13], although a diminished chondrogenic potential of the magnetically labeled stem cells was observed [14]. Several SPION formulations composed of magnetite/maghemite (Fe_3_O_4_/Fe_2_O_3_), coated with dextran (Feridex^®^) or carboxydextran (Resovist^®^), have been approved for the clinic [15,16].

A unique property of SPION is the efficient generation of heat when exposed to an alternating magnetic field (AMF), which can be used for therapeutic applications [17]. Mechanical forces generated by the interaction of SPION with a gradient magnetic field have also been used for multiple applications, including magnetic tweezers, nanosensing, magnetic cell separation, specific delivery of genes and therapeutic agents, and mechanical modulation in cells [5,6,18,19,20,21,22] or tumor models [23]. Low-strength magnetic fields have also been used to destroy human tumor cells with polymer-coated, multi-walled carbon nanotubes [24]. The effect of AMF on the survivability of cells labeled with MNP without a temperature increase has also been reported [25,26,27].

Here, we demonstrate a new strategy for the destruction of MNP-labeled cells by exposing them to oscillating gradients of a magnetic field in the presence of a static saturating magnetic field. In this report, we evaluate this method *in vitro* in cultured triple-negative breast cancer MDA-MB-231 cells. We hypothesize that the mechanism of cell destruction is mediated by direct mechanical forces generated by the magnetic interaction of the MNP aggregates with the gradient field, and is not related to AMF-induced hyperthermia. Therefore, this technique should selectively destroy targeted MNP-labeled cells with minimal effect on neighboring unlabeled cells.

## Materials and Methods

### Nanoparticles

For this study, Bionized NanoFerrite (BNF) superparamagnetic iron oxide MNP, coated with starch (plain surface, 80 nm diameter), were purchased from Micromod Partikeltechnologie GmbH, Rostock, Germany, and used without further modification. The stock solution has an iron concentration of 13.7 mg/ml, and BNF MNP have a typical mass magnetization of 49 A m^2^/kg Fe at 79,500 A/m; a saturation magnetization μ_sat_ > 76 A m^2^/kg Fe at magnetic field H > 7.95•10^5^ A/m; and the coercive field Hc = 449 A/m.

### Pulse sequence

**Fig 1:**
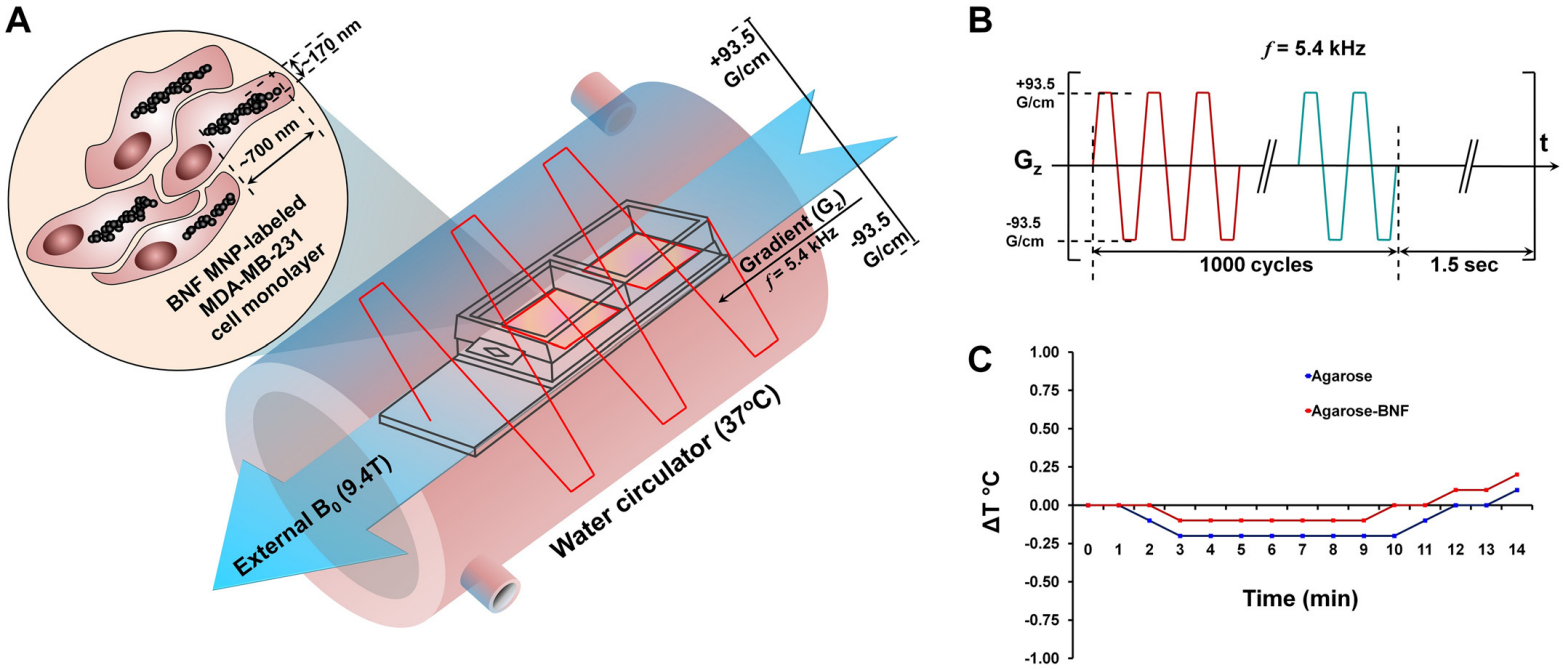
Specifications of the treatment and the gradient system. **(A)** Schematic diagram of the therapeutic system. **(B)** Gradient pulse sequence used in the high magnetic field. **(C)** Changes in local temperatures in agarose samples prepared with (100 μg/ml) and without MNP.

Fig 1A illustrates the experimental setup in a high magnetic field B_0_ = 9.4T of a preclinical MRI system. A gradient pulse sequence shown in Fig 1B was developed, using the Paravision programming environment and installed on a 9.4T Bruker Biospec system equipped with a G060 gradient system (60 mm inner diameter, 95 G/cm maximum gradient strength, and 50 μs rise time). The gradient sequence, which generated an oscillating G_z_ gradient, was applied to the samples for approximately 60 min, with a duty cycle of 7%. The thermal effect of the treatment was studied in agarose samples prepared in saline (0.9% NaCl in purified H_2_O) with and without MNP (100 μg/mL), using an immersed thermocouple probe. The samples were positioned in a circulating water chamber with the temperature set at 37°C. The temperature changes in MNP-agarose samples were compared with agarose controls without MNP (Fig 1C).

### Cancer cells

**Fig 2:**
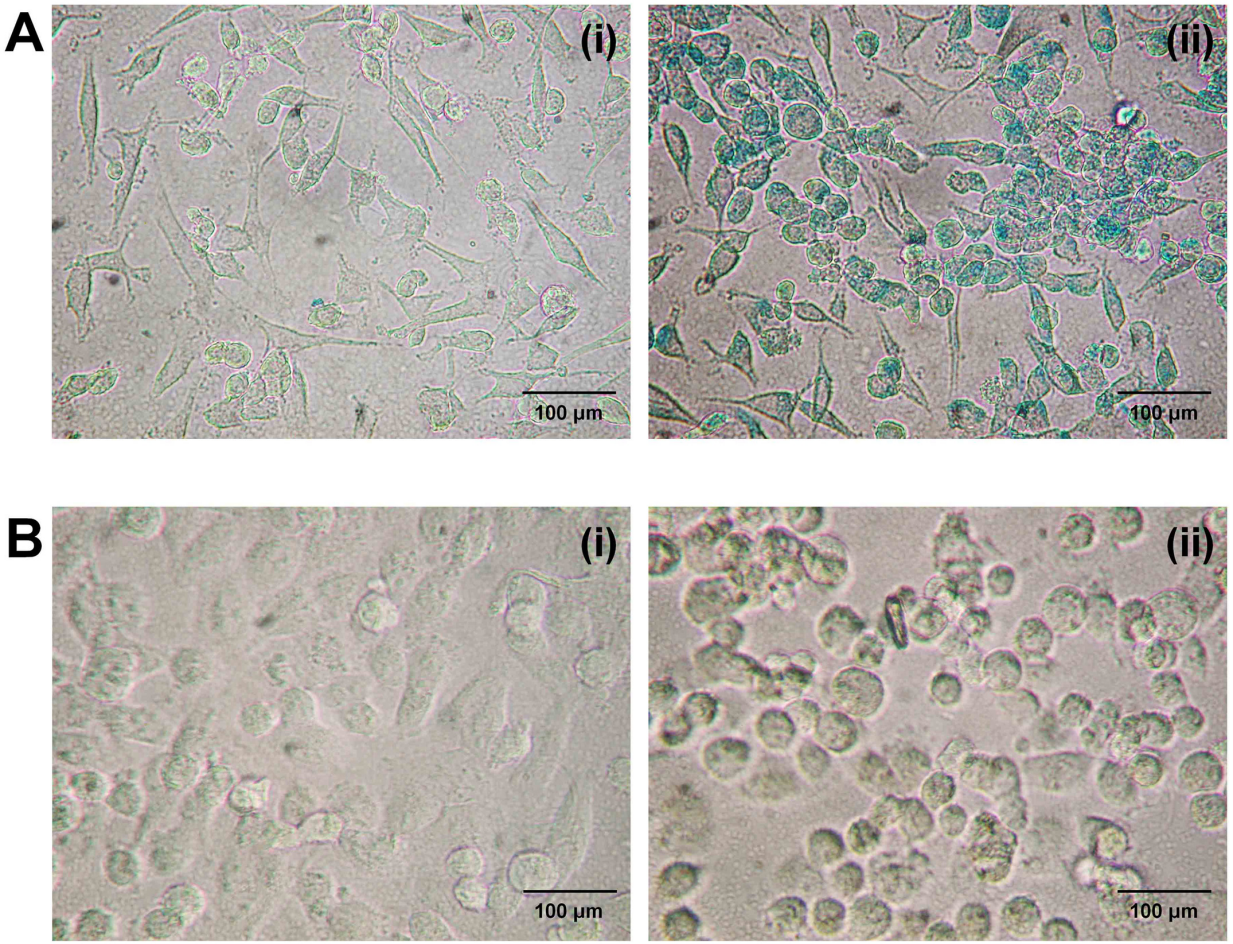
MNP-labeling and the therapeutic effect in MDA-MB-231cells. **(A)** Prussian blue staining of unlabeled (i) and MNP-labeled (ii) cells. **(B)** MNP-labeled cells before treatment (i) and immediately after the treatment (ii).

Human breast carcinoma MDA-MB-231 cells (ATCC) were cultured in DMEM (Cellgro) medium supplemented with 1% penicillin-streptomycin and 10% FBS, and maintained at 37°C in a humidified atmosphere containing 5% CO_2_ unless otherwise mentioned. Third or fourth passages of cells with 70–80% confluency were used for imaging and therapeutic experiments. Cells were seeded in four-well chamber slides (1×10^5^ cells/well), grown for 24 h to ~75% confluency, and were labeled with MNP following an established protocol [28]. Briefly, 9 μL of MNP (27.4 mg/mL) was gently stirred with 2.5 μL poly-l-lysine (PLL, 1.5 mg/mL) in 10 mL of cell culture media at room temperature for 1 h, for a final MNP concentration of 25 μg/mL, which resulted in the formation of physically bound MNP-PLL complexes. In this study, cells were incubated in this media for 24 h at 37°C and rinsed thoroughly with PBS, and the dishes were supplied with fresh media. The cell labeling was confirmed by Prussian blue staining (Fig 2A). Based on inductively coupled plasma mass spectrometric analysis (ICP-MS), this method results in an iron upload per cell of 14.8 ± 1.7 pg [11,29].

### Stability of nanoparticles

Chemical stability of MNPs and their starch-coating were studied by measuring the hydrodynamic diameter of the particles (MNP 25.0 μg/mL in DMEM) using dynamic light scattering (DLS) MALVERN Nano ZS90 Zetasizer before and after the exposure to oscillating gradients.

### Effect of the oscillating gradients on MNP-labeled cancer cells

#### LIVE/DEAD^®^ cell imaging

The viability of cells after 24 h post exposure to the gradients in the horizontal bore magnet of the 9.4T Bruker MRI spectrometer was qualitatively analyzed by LIVE/DEAD^®^ (Life Technologies, Inc.) in the cell microscopy experiments. In this study, MNP-labeled or unlabeled MDA-MB-231 cells grown in four-well chamber slides were exposed to the gradient treatment, as described above, and incubated for 24 h. The media were replaced by LIVE/DEAD^®^ cell imaging mixture following the manufacturer's protocol. The cell cultures were incubated for 20 min and imaged by fluorescence microscope using green (live cells) and red (dead cells) channels.

#### MTS assay

The viability of cells after the gradient treatment was quantitatively analyzed by MTS assay. The MDA-MB-231 cells in four-well chamber slides were exposed to the gradient treatment and incubated for 24 h. The media was replaced with 10% MTS in medium and incubated for two hours. The absorbance of the media was measured at 490 nm. The percentages of dead cells were calculated with respect to the viable cell count in the unlabeled and untreated cell population.

### Transmission electron microscopy (TEM)

TEM was used to study the alignment of internalized MNP along the magnetic field. The MNP-labeled cells were placed in the bore of a 4.7T Bruker MRI spectrometer (inner diameter of 40 cm) for 60 min, to induce an alignment of the internalized MNP along the magnetic field. The large bore of the magnet allowed manipulations with the cells while in the magnetic field. Following this, the cells were fixed while still in the magnet, using a 0.1 M sodium cacodylate buffer solution (pH 7.2) containing 2.5% glutaraldehyde, 3 mM CaCl_2_, and 1% sucrose, for one hour. Next, the cells were rinsed, three times, with a 0.1 M sodium cacodylate buffer solution (pH 7.2) for 15 min. The cellular lipid membranes were then fixed with a 1% potassium permanganate solution for 30 min, on ice, and in the dark. The cells were next rinsed with deionized water, dehydrated in a graded series of ethanol, and embedded in an Eponate 12 resin (Ted Pella Incorporated, Redding, CA, USA). Following this, the samples were polymerized while still in the magnet at 37°C for 48 h to preserve the orientation and structure of the internalized MNP. The dishes were maintained in the same position and orientation within the magnet, throughout the whole process. The rigid samples were then removed from the magnet and further polymerized at 60°C for 24 h. Following the polymerization step, thin sections (60 to 90 nm) were cut with a diamond knife on the Reichert-Jung Ultracut E ultramicrotome and picked up with naked 200-mesh copper grids. Grids were next observed on a Philips CM120 TEM at 80 kV.

### Optical microscopy

For optical microscopy studies, MDA-MB-231 cells were grown to ~80% confluency, in four-well chamber slides. The cells were then labeled with MNP as described above, rinsed thoroughly with PBS, and fresh media was placed in the wells. The cells were then placed in the bore of a 9.4T Bruker MRI spectrometer for 30 min, and the fixation process was carried out as described above. However, following the dehydration step, the polymerization step was omitted and the slides were mounted with a Permount mounting medium (Fisher Scientific, Pittsburgh, PA) while still in the magnet. The orientation of the chamber slides in the magnet was maintained throughout the process. Following this, samples were imaged using a Nikon Eclipse TS100 microscope.

Rearrangement and alignment dynamics of MNP aggregates were also studied in living MDA-MB-231 cells grown and labeled with MNP, as described above. Cells in four-well microscopy chamber slides were positioned inside a 9.4T MRI magnet at 37°C for a variable amount of time, and light microscopy was performed immediately after B_0_ exposure using an inverted microscope with 40x lens. Extreme care was used to slowly load and remove the samples from the magnet bore parallel to the magnet axis, while maintaining close proximity to the axis to prevent possible changes in the cluster orientation due to magnetic torque. Images were converted to 16-bit gray scale and processed with NIH ImageJ software to derive the directionality parameter that reports the preferred orientation of structures present in the input image using a standard ImageJ directionality plugin. Briefly, this plugin calculates the preferred orientation of structures present in the image. It computes a histogram that indicates the relative number of structures in a given direction [30,31]. Optical microscopy images were acquired before and after 2, 5, 10, 20, 30, 45, and 60 minutes exposure to the magnetic field.

### Statistical analysis

Triplicate independent experiments were carried out for the statistical analyses. A two-tailed Student’s *t*-test was used to analyze changes in cell viability. The difference was considered significant when the *p*-value was <0.05.

## Results

**Fig 3:**
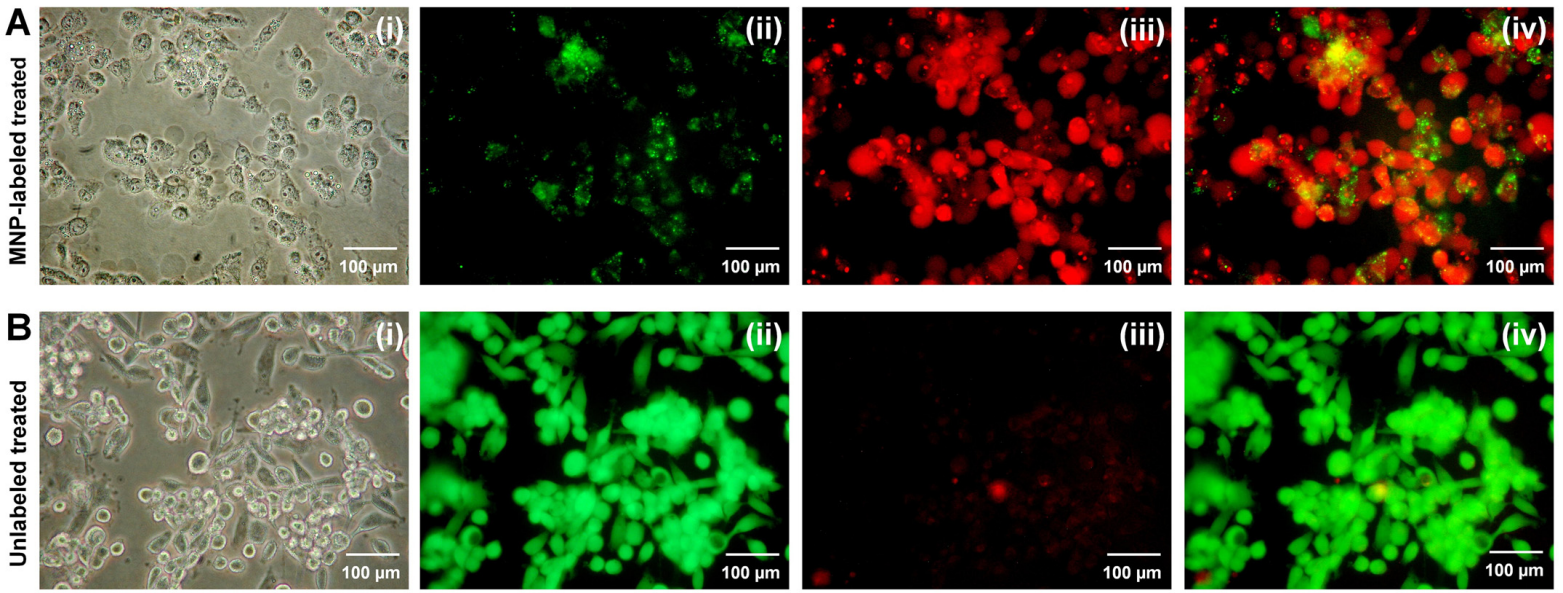
Effects of treatment on cancer cells assessed by LIVE/DEAD^®^ assay microscopy in MDA-MB-231 cells. (A) LIVE/DEAD^®^ cell microscopic images of MNP-labeled, treated cells after 24 h. (i) Phase contrast optical image, (ii) distribution of live cells, (iii) distribution of dead cells, and (iv) merged image. (B) LIVE/DEAD^®^ cell microscopic images of unlabeled, treated cells after 24 h. (i) Phase contrast optical image, (ii) distribution of live cells, (iii) distribution of dead cells, and (iv) merged image.

Cell labeling was confirmed by Prussian blue staining and the cells remained healthy and showed no change in morphology (Fig 2) before the treatment. According to the Li *et al*. at least 400 μg/mL of uncoated MNP with 24 h incubation is required for significant cytotoxicity in cancer cells [32]. We have incubated cells in this media containing 25 μg/ml MNP-PLL complexes up to 5 days and observed no MNP cytotoxicity. Immediately after the gradient treatment, a significant amount of labeled cells were detached from the chamber surface and the morphology of the cells that remained attached was significantly altered (Fig 2B). The maximum cell damage was observed for the highest gradient switching frequency, *f*, allowed by the hardware (*f* ~ 5.4 kHz). The LIVE/DEAD^®^ cell microscopy assay showed a significant amount of dead cells in the MNP-labeled and treated cell population compared to the unlabeled treated cell population at 24 h after the treatment (Fig 3). Unlabeled, gradient treated, and MNP-labeled, untreated MDA-MB-231 cells were used as controls for all studies, and no cytotoxicity to the control MDA-MB-231 cells was observed as shown in S1 Appendix. No increase in temperature was detected during treatment in the MNP-agarose sample, or in the agarose not containing MNP (Fig 1C). Therefore, the detected cellular effects were likely caused by the direct mechanical action of MNP and not by a thermal effect during the treatment. In addition, we observed no change in hydrodynamic diameter of the particles before and after the gradient treatments (S2 Appendix). Therefore, the observed cellular effects cannot be related to potential toxicity of uncoated iron-oxide nanoparticles.

**Fig 4:**
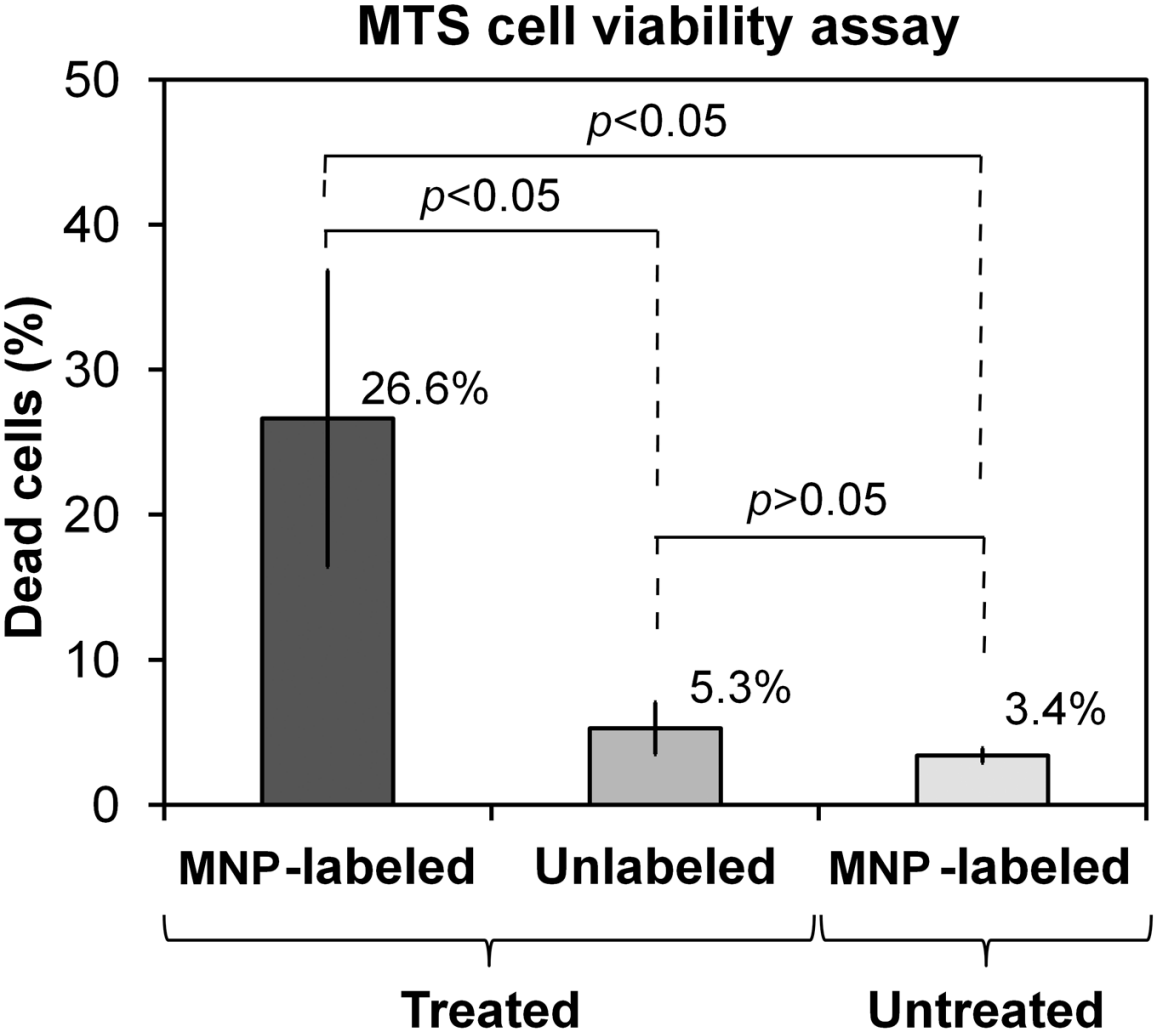
MTS cell viability assay. The percentage of dead cells in MNP-labeled/treated, unlabeled/treated, and MNP-labeled/untreated MDA-MB-231 cells was measured with respect to the unlabeled/untreated cell population.

Viability of MDA-MB-231 cells after the treatment was also evaluated by MTS assay (Fig 4). We observed 26.6% of dead cells in the MNP-labeled, treated cell population after 24 h of the treatment. The percentages of dead cells in unlabeled/treated cells and MNP-labeled/untreated cells were 5.3% (p<0.05) and 3.4% (p<0.05), respectively. The percentages of dead cells were calculated with respect to the cell population on unlabeled/untreated samples. Changes in cell viability were considered statistically significant for *p* values less than 0.05.

**Fig 5:**
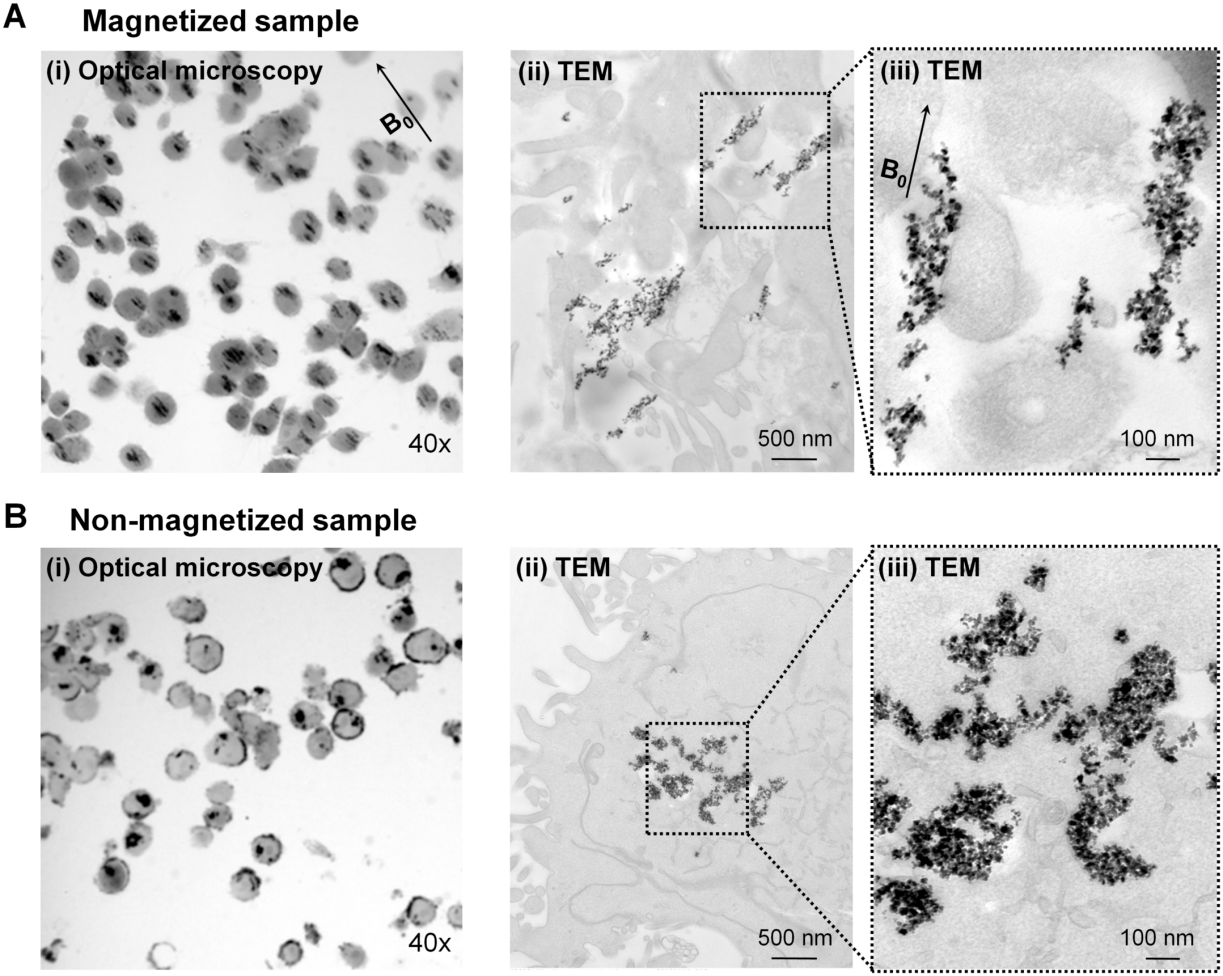
Microscopic images of MNP aggregates orientation in MDA-MB-231 cells. Optical microscopy and TEM micrographs of the orientation of MDA-MB-231 cells incubated **(A)** at B_0_ = 4.7T magnetic field (i) optical image at 40x, (ii) TEM at 17,500x (scale bar, 500 nm), and (iii) TEM at 65,000x (scale bar, 100 nm), or **(B)** at a non-magnetic condition (i) optical image at 40x, (ii) TEM at 17,500x (scale bar, 500 nm), and (iii) TEM at 65,000x (scale bar, 100 nm).

TEM analysis of the MNP-labeled cells, maintained at a 4.7T external magnetic field, revealed the formation of elongated MNP structures with diameters of ~170 nm and lengths of ~700 nm (Fig 5A), compared to the samples not exposed to the magnetic field (Fig 5B). These structures consisted of 250–300 MNP particles primarily found in late endosomal compartments, and were oriented parallel to the B_0_ magnetic field and applied G_z_ gradients. Optical images (using Nikon Eclipse TS100 microscope CCD camera, and processed by NIH ImageJ software) of the labeled cells demonstrated that the MNP structures could also be resolved by optical microscopy.

**Fig 6:**
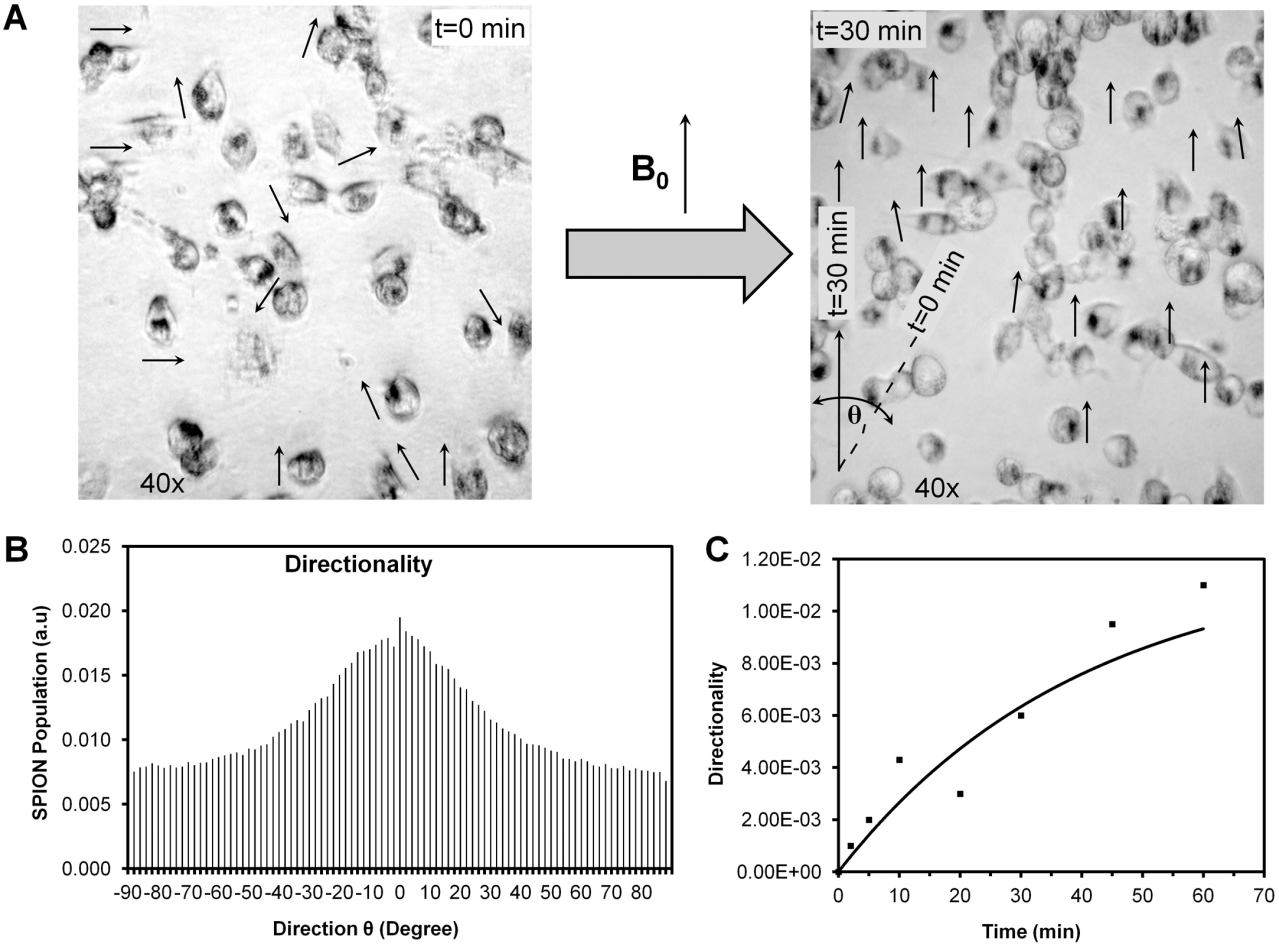
Magnetic directionality of MNP aggregates. **(A)** Example of images used to evaluate the magnetic directionality of MNP aggregates in MDA-MB-231 cells at *t = 0* and *t = 30* min after exposure to the external magnetic field (B_0_ = 9.4T). **(B)** Directionality histogram at *t = 30* min. **(C)** Change in directionality (a.u.) with magnetization time, *t* (min).

Microscopic images of MNP-labeled cells obtained before and after exposure to the 9.4T field (37°C, 30 min, using a Bruker Biospec 9.4T preclinical MRI system) are shown in Fig 6A. A directionality histogram is presented in Fig 6B, and changes in the directionality parameter at angle 0° (parallel to magnetic field) as a function of magnetic exposure time are shown in Fig 6C. A typical orientation rearrangement time of 39 min in the 9.4T magnet was observed (*R*^2^ = 0.92).

## Discussion

The observed cell destruction by the gradient treatment was attributed to the interaction of magnetized MNP aggregates with external gradient fields. A detailed description of the mechanical effects of the combination of the static and gradient magnetic fields on nanoparticles was reported by Carrey et al. [6]. Briefly, in our experiments, the magnetic field (*B*_*0*_) saturated the magnetic core of the nanoparticle to its maximum value of μ_*sat*_. At this condition, the force on the nanoparticle produced by the gradient, *dB*_*0*_*/dR*, can be derived as

$$F=\frac{1}{2}\nabla \left(m_{sat}B_{0}\right)=\frac{V}{2}M_{sat}\frac{dB_{0}}{dR}$$ (1)

where *V* is the particle volume and *M*_*sat*_ is the volumetric saturated magnetization [33] (S3 Appendix). A simple estimation of the magnetic force for a single MNP with a diameter of 50 nm and a saturating magnetization *μ*_*0*_*M*_*sat*_ of ~1T, positioned in a gradient magnetic field *G = dB/dR = 0*.*5 T/m*, results in *F* ≈ 10^−17^ N. This force is significantly lower than the force required to destroy the cellular membrane [6]. Therefore, only a synchronous action of magnetic aggregates, consisting of multiple MNP, magnetized by the B_0_ field and driven by audio frequency gradients, can produce efficient cell damage. In this case, an increase of the mechanical force by at least several orders of magnitude is expected due to the increased volume and orientation of the elongated MNP aggregates along the magnetic field and the applied gradients. The relatively low frequency of the applied gradients, *f*, suggests the stationary regime for MNP interaction with the magnetic field *1/f* >> *τ = m/K*_*f*,_ where *m* is the mass of MNP and *K*_*f*_ is the friction force of the medium with viscosity *η*(*Kf = 6πηR*) [6]. It is also quite likely that the free drift of the MNP aggregates that are internalized in the cell is limited by the internal membranes of the cellular compartments, such as endosomes and lysosomes. Significantly enhanced effects of cell destruction were detected for the chosen direction of gradients, G_z_, parallel to the magnetic field. This orientation induces motion of the aggregates along their long axis with minimal resistance from the medium, which results in the increased amplitude of the motion and presumably enhanced cell effects. Importantly, the alternating magnetic field in this case should produce the highest effect after the alignment of the magnetic aggregates with the B_0_ is complete, as was ensured in our experiments.

Therefore, the major effects of the static magnetic field B_0_, which are critically important for the cellular effects from the oscillating gradients are: (i) saturation of the MNP magnetization, which maximizes the magnetic force applied to the MNP Eq (1); and (ii) formation of supramolecular aggregates that consist of multiple MNP, which significantly amplifies the magnetic forces compared to the force on a single magnetic nanoparticle.

Other possible mechanisms of cell damage due to the interaction of the oscillating magnetic field with MNP-labeled cells, such as hysteresis, induction heating by eddy currents, and ferromagnetic resonance, are described in S4 Appendix and reveal negligible effects of heating in magnetically saturated MNP or conductive cell growth media, as has been confirmed by our control experiments. The iron content of the labeled cell was significantly lower than the cytotoxic level of coated or uncoated MNP in cancer cells [32]. The starch-coating of the nanoparticle is chemically cross-linked and the gradient treatment, which only induces bulk motion of the nanoparticle-assemblies, does not destroy the coating.

Selective cell kill by rotational movement of magnetic nanoparticles attached to the lysosomal membrane via anti-LAMP1 antibodies was demonstrated by Zhang et al. [34]. Low-frequency (30 mT, 20 Hz) dynamic magnetic fields induced rotation of the 100 nm diameter LAMP1-SPION and apparently apoptotic cell death due to the tearing of the lysosomal membrane [34]. However, in this approach, MNP were not magnetically saturated by the applied magnetic field and did not form aggregates that could generate significantly amplified mechanical torque and forces compared to individual magnetic nanoparticles. Indeed, the analysis in Carrey et al. suggests that an MNP with a diameter in the micron range is required to produce mechanical forces in the piconewton (10^−12^ N) range when exposed to the gradient of a magnetic field of *G = 1 T/m* [6]. Lethal damage to the cell membrane produced by a monoclonal antibody conjugated with the photosensitizing phthalocyanine dye, IRDye 700DX, has also been related to mechanical effects on the cell membrane [35,36].

## Conclusion

Overall, our results demonstrate that oscillating gradients can selectively destroy MNP-labeled cells positioned in a saturated magnetic field. The technique is not based on MNP-induced hyperthermia, and we suggest that the effect is due to mechanical forces generated by internalized MNP aggregates. It is important to note that, for an external magnetic field B_0_ that is above the saturation field, the magnetic force does not explicitly depend on B_0_ and the method should provide a similar efficacy for B_0_ ≥ ~1.5T, which is the typical range for clinical MRI. This report takes an important first step toward future multiple biomedical applications, including the destruction of cancer and transplanted cells. In addition, MNP-labeled cells generate strong MRI contrast, which facilitates image-guided applications for this method.

## Supporting Information

S1 Appendix LIVE/DEAD^®^ cell images of controls. **(A)** LIVE/DEAD^®^ cell assay on unlabeled and magnetically treated cells. **(B)** LIVE/DEAD^®^ cell assay on MNP-labeled and untreated cells exposed to the static magnetic field B_0_ only.
(PDF)

S2 Appendix Stability of MNPs during the gradient treatment. Stability of the MNPs and their starch-coating were studied by measuring the hydrodynamic diameter.
(PDF)

S3 Appendix Magnetic forces. The magnetic force generated by a gradient magnetic field on MNP.
(PDF)

S4 Appendix Heating effects. The heating of conductive MNP in variable magnetic field.
(PDF)

## References

[1] Penet MF, Artemov D, Farahani K, Bhujwalla ZM (2013) MR—eyes for cancer: looking within an impenetrable disease. Nmr in Biomedicine 26: 745–755. 10.1002/nbm.2980 23784955 PMC3690531

[2] Shapiro EM, Skrtic S, Sharer K, Hill JM, Dunbar CE, Koretsky AP (2004) MRI detection of single particles for cellular imaging. Proceedings of the National Academy of Sciences of the United States of America 101: 10901–10906. 15256592 10.1073/pnas.0403918101 PMC503717

[3] Chen CCV, Ku MC, Jayaseema DM, Lai JS, Hueng DY, Chang C (2013) Simple SPION Incubation as an Efficient Intracellular Labeling Method for Tracking Neural Progenitor Cells Using MRI. Plos One 8. 10.1371/journal.pone.0056125 PMC3585319 23468856

[4] Ivkov R, DeNardo SJ, Daum W, Foreman AR, Goldstein RC, Nemkov VS, et al (2005) Application of high amplitude alternating magnetic fields for heat induction of nanoparticles localized in cancer. Clinical Cancer Research 11: 7093s–7103s. 16203808 10.1158/1078-0432.CCR-1004-0016

[5] Tseng P, Judy JW, Di Carlo D (2012) Magnetic nanoparticle-mediated massively parallel mechanical modulation of single-cell behavior. Nature Methods 9: 1113-+ 10.1038/nmeth.2210 23064517 PMC3501759

[6] Carrey J, Connord V, Respaud M (2013) Ultrasound generation and high-frequency motion of magnetic nanoparticles in an alternating magnetic field: Toward intracellular ultrasound therapy? Applied Physics Letters 102.

[7] Frank JA, Miller BR, Arbab AS, Zywicke HA, Jordan EK, Lewis BK, et al (2003) Clinically applicable labeling of mammalian and stem cells by combining superparamagnetic iron oxides and transfection agents (vol 228, pg 480, 2003). Radiology 229: 610–610. 12819345 10.1148/radiol.2281020638

[8] Kraitchman DL, Kedziorek DA, Bulte JWM (2011) MR Imaging of Transplanted Stem Cells in Myocardial Infarction. Moleuclar Imaging: Methods and Protocols 680: 141–152. 10.1007/978-1-60761-901-7_10 PMC3076084 21153379

[9] Willenbrock S, Knippenberg S, Meier M, Hass R, Wefstaedt P, Nolte I, et al (2012) In Vivo MRI of Intraspinally Injected SPIO-labelled Human CD34(+) Cells in a Transgenic Mouse Model of ALS. In Vivo 26: 31–38. 22210713

[10] Bigini P, Diana V, Barbera S, Fumagalli E, Micotti E, Sitia L, et al (2012) Longitudinal Tracking of Human Fetal Cells Labeled with Super Paramagnetic Iron Oxide Nanoparticles in the Brain of Mice with Motor Neuron Disease. Plos One 7. 10.1371/journal.pone.0032326 PMC3288077 22384217

[11] Ngen EJ, Wang L, Kato Y, Krishnamachary B, Zhu W, Gandhi N, et al (2015) Imaging transplanted stem cells in real time using an MRI dual-contrast method. Sci Rep 5: 13628 10.1038/srep13628 26330231 PMC4556978

[12] Bulte JWM (2009) In Vivo MRI Cell Tracking: Clinical Studies. American Journal of Roentgenology 193: 314–325. 10.2214/AJR.09.3107 19620426 PMC2857985

[13] Schafer R, Bantleon R, Kehlbach R, Siegel G, Wiskirchen J, Wolburg H, et al (2010) Functional investigations on human mesenchymal stem cells exposed to magnetic fields and labeled with clinically approved iron nanoparticles. Bmc Cell Biology 11. 10.1186/1471-2121-11-22 PMC2871263 20370915

[14] Henning TD, Sutton EJ, Kim A, Golovko D, Horvai A, Ackerman L, et al (2009) The influence of ferucarbotran on the chondrogenesis of human mesenchymal stem cells. Contrast Media Mol Imaging 4: 165–173. 10.1002/cmmi.276 19670250 PMC2933782

[15] Reimer P, Rummeny EJ, Daldrup HE, Balzer T, Tombach B, Berns T, et al (1995) Clinical-Results with Resovist—a Phase-2 Clinical-Trial. Radiology 195: 489–496. 7724772 10.1148/radiology.195.2.7724772

[16] Wang YX (2011) Superparamagnetic iron oxide based MRI contrast agents: Current status of clinical application. Quant Imaging Med Surg 1: 35–40. 10.3978/j.issn.2223-4292.2011.08.03 23256052 PMC3496483

[17] Kut C, Zhang Y, Hedayati M, Zhou H, Cornejo C, Bordelon D, et al (2012) Preliminary study of injury from heating systemically delivered, nontargeted dextran-superparamagnetic iron oxide nanoparticles in mice. Nanomedicine 7: 1697–1711. 10.2217/nnm.12.65 22830502 PMC3991127

[18] Colombo M, Carregal-Romero S, Casula MF, Gutierrez L, Morales MP, Bohm IB, et al (2012) Biological applications of magnetic nanoparticles. Chemical Society Reviews 41: 4306–4334. 10.1039/c2cs15337h 22481569

[19] Pankhurst QA, Thanh NTK, Jones SK, Dobson J (2009) Progress in applications of magnetic nanoparticles in biomedicine. Journal of Physics D-Applied Physics 42.

[20] Swierczewska M, Liu G, Lee S, Chen XY (2012) High-sensitivity nanosensors for biomarker detection. Chemical Society Reviews 41: 2641–2655. 10.1039/c1cs15238f 22187721 PMC3629948

[21] Zhang E, Kircher MF, Koch M, Eliasson L, Goldberg SN, Renstrom E (2014) Dynamic magnetic fields remote-control apoptosis via nanoparticle rotation. Acs Nano 8: 3192–3201. 10.1021/nn406302j 24597847 PMC4004315

[22] Kim DH, Rozhkova EA, Ulasov IV, Bader SD, Rajh T, Lesniak MS, et al (2010) Biofunctionalized magnetic-vortex microdiscs for targeted cancer-cell destruction. Nat Mater 9: 165–171. 10.1038/nmat2591 19946279 PMC2810356

[23] Bouchlaka MN, Sckisel GD, Wilkins D, Maverakis E, Monjazeb AM, Fung M, et al (2012) Mechanical disruption of tumors by iron particles and magnetic field application results in increased anti-tumor immune responses. Plos One 7: e48049 10.1371/journal.pone.0048049 23133545 PMC3485005

[24] Liu D, Wang L, Wang Z, Cuschieri A (2012) Magnetoporation and magnetolysis of cancer cells via carbon nanotubes induced by rotating magnetic fields. Nano Lett 12: 5117–5121. 10.1021/nl301928z 22950948

[25] Creixell M, Bohorquez AC, Torres-Lugo M, Rinaldi C (2011) EGFR-targeted magnetic nanoparticle heaters kill cancer cells without a perceptible temperature rise. Acs Nano 5: 7124–7129. 10.1021/nn201822b 21838221

[26] Domenech M, Marrero-Berrios I, Torres-Lugo M, Rinaldi C (2013) Lysosomal membrane permeabilization by targeted magnetic nanoparticles in alternating magnetic fields. Acs Nano 7: 5091–5101. 10.1021/nn4007048 23705969

[27] Sanchez C, El Hajj Diab D, Connord V, Clerc P, Meunier E, Pipy B, et al (2014) Targeting a G-protein-coupled receptor overexpressed in endocrine tumors by magnetic nanoparticles to induce cell death. ACS Nano 8: 1350–1363. 10.1021/nn404954s 24401079

[28] Kraitchman DL, Kedziorek DA, Bulte JW (2011) MR imaging of transplanted stem cells in myocardial infarction. Methods Mol Biol 680: 141–152. 10.1007/978-1-60761-901-7_10 21153379 PMC3076084

[29] Bulte JW, Kraitchman DL (2004) Iron oxide MR contrast agents for molecular and cellular imaging. NMR Biomed 17: 484–499. 15526347 10.1002/nbm.924

[30] Liu ZQ (1991) Scale Space Approach to Directional Analysis of Images. Applied Optics 30: 1369–1373. 10.1364/AO.30.001369 20700292

[31] Woolley AJ, Desai HA, Steckbeck MA, Patel NK, Otto KJ (2011) In situ characterization of the brain-microdevice interface using Device Capture Histology. Journal of Neuroscience Methods 201: 67–77. 10.1016/j.jneumeth.2011.07.012 21802446 PMC3179652

[32] Li L, Mak KY, Shi J, Koon HK, Leung CH, Wong CM, et al (2012) Comparative In Vitro Cytotoxicity Study on Uncoated Magnetic Nanoparticles: Effects on Cell Viability, Cell Morphology, and Cellular Uptake. Journal of Nanoscience and Nanotechnology 12: 9010–9017. 23447952 10.1166/jnn.2012.6755

[33] Pankhurst QA, Connolly J, Jones SK, Dobson J (2003) Applications of magnetic nanoparticles in biomedicine. Journal of Physics D-Applied Physics 36: R167–R181.

[34] Zhang EM, Kircher MF, Koch M, Eliasson L, Goldberg SN, Renstrom E (2014) Dynamic Magnetic Fields Remote-Control Apoptosis via Nanoparticle Rotation. Acs Nano 8: 3192–3201. 10.1021/nn406302j 24597847 PMC4004315

[35] Mitsunaga M, Ogawa M, Kosaka N, Rosenblum LT, Choyke PL, Kobayashi H (2011) Cancer cell-selective in vivo near infrared photoimmunotherapy targeting specific membrane molecules. Nat Med 17: 1685–1691. 10.1038/nm.2554 22057348 PMC3233641

[36] Mitsunaga M, Nakajima T, Sano K, Kramer-Marek G, Choyke PL, Kobayashi H (2012) Immediate in vivo target-specific cancer cell death after near infrared photoimmunotherapy. BMC Cancer 12: 345 10.1186/1471-2407-12-345 22873679 PMC3502522

