# Supplementary material for: Non-Temperature Induced Effects of Magnetized Iron Oxide Nanoparticles in Alternating Magnetic Field in Cancer Cells
Source: PLoS One. 2016 May 31;11(5):e0156294. doi: 10.1371/journal.pone.0156294 (PMC4887104; doi:10.1371/journal.pone.0156294)
Supplement: S1 Appendix — (A) LIVE/DEAD® cell assay on unlabeled and magnetically treated cells. (B) LIVE/DEAD® cell assay on MNP-labeled and untreated cells exposed to the static magnetic field B0 only. (PDF) [file pone.0156294.s001.pdf]

*Supporting information*

**S1 Appendix. LIVE/DEAD® cell images of controls.**

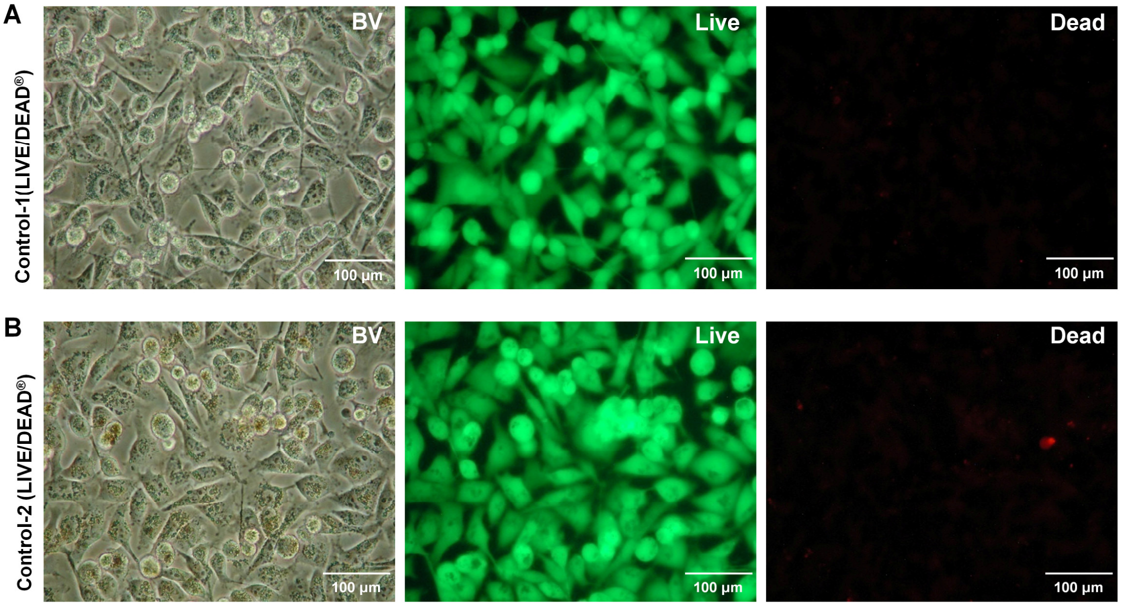


LIVE/DEAD® cell images of controls. **(A)** LIVE/DEAD® cell assay on unlabeled and magnetically treated cells. **(B)** LIVE/DEAD® cell assay on MNP-labeled and untreated cells exposed to the static magnetic field $B_0$ only.
