# Supplementary material for: Non-Temperature Induced Effects of Magnetized Iron Oxide Nanoparticles in Alternating Magnetic Field in Cancer Cells
Source: PLoS One. 2016 May 31;11(5):e0156294. doi: 10.1371/journal.pone.0156294 (PMC4887104; doi:10.1371/journal.pone.0156294)

Supporting information

S2 Appendix. Stability of MNPs during the gradient treatment

Stability of the MNPs and their starch-coating were studied by measuring the hydrodynamic diameter of the particles using dynamic light scattering (DLS) MALVERN Nano ZS90 Zetasizer. The hydrodynamic diameters of MNPs in the medium before and after the gradient treatment was measured as 120 ± 2.1 nm and 119 ± 2.3 nm, respectively. No significant difference was detected in these studies, which confirms the stability of BNF nanoparticles throughout the procedure.

(A) Before the gradient treatment (120 ± 2.1 nm)

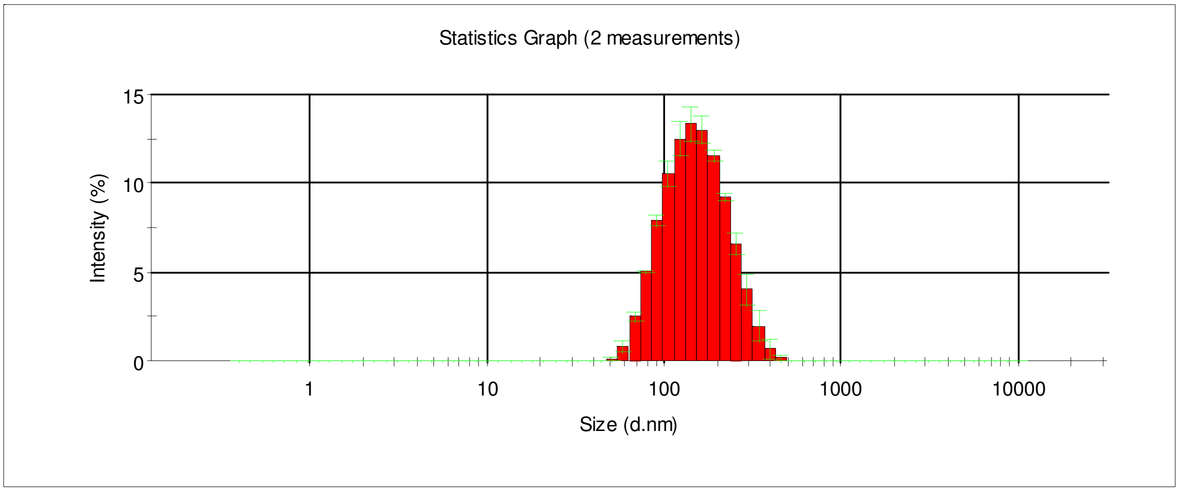

(B) After the gradient treatment (119 ± 2.3 nm)

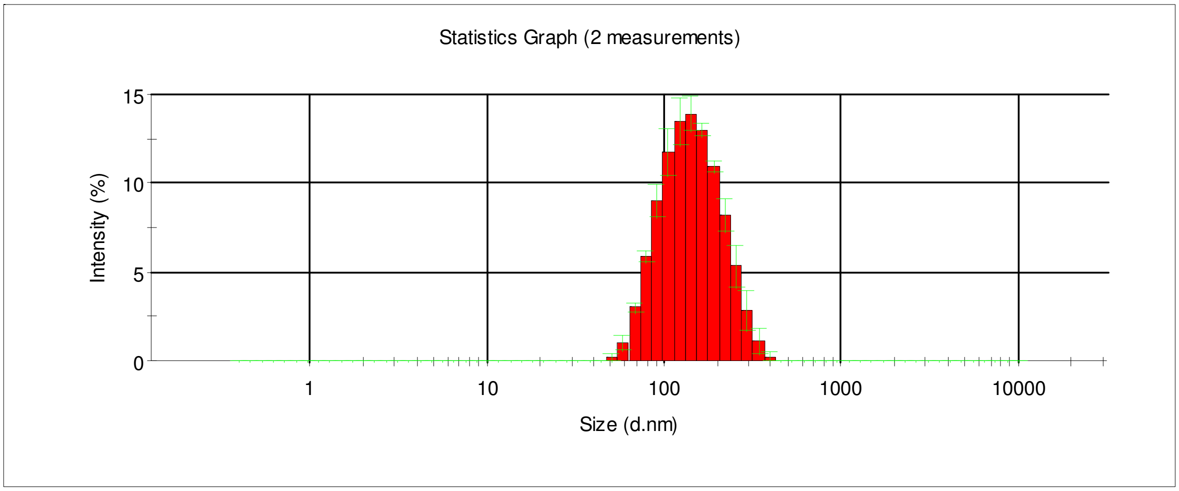

Supplement: S2 Appendix — Stability of the MNPs and their starch-coating were studied by measuring the hydrodynamic diameter. (PDF) [file pone.0156294.s002.pdf]
