# Supplementary material for: Non-Temperature Induced Effects of Magnetized Iron Oxide Nanoparticles in Alternating Magnetic Field in Cancer Cells
Source: PLoS One. 2016 May 31;11(5):e0156294. doi: 10.1371/journal.pone.0156294 (PMC4887104; doi:10.1371/journal.pone.0156294)
Supplement: S3 Appendix — The magnetic force generated by a gradient magnetic field on MNP. (PDF) [file pone.0156294.s003.pdf]

S3 Appendix. Magnetic forces

The magnetic force generated by a gradient magnetic field on a spherical magnetic particle is given by equation (1):

$$F = \nabla(U) = -1/2\nabla(m \cdot B_0), \qquad (1)$$

where m is the magnetic moment of the sphere defined as $m=V\bullet M$, where V is the particle volume and M is volumetric magnetization. The magnetization of the MNP depends on the particle material and magnetization conditions. For a paramagnetic (superparamagnetic) particle in a weak magnetic field, $B=H,$ the magnetization is given by $M=\Delta\chi\ H,$ where $\Delta\chi=\chi_m\text{-}\chi_w$ and χ_m and χ_w are the magnetic susceptibility of the particle and the medium (water), respectively. A spherical ferromagnetic particle in a weak magnetic field, $B=\mu_0 H,$ has the magnetization $M=3H,$ and, considering the demagnetizing factor of 1/3 for a sphere, the magnetic field in the sphere is $B = \mu_0 H + \mu_0 M = B_0 - 1/3\bullet\mu_0 M + \mu_0 M = B_0 + 2/3\bullet\mu_0 M \approx 3\ B_0$. If the external magnetic field is above the saturation field for the magnetic material of MNP, the particle magnetization is $M=M_{sat}=const$ and the magnetic field $B = B_0 + 2/3\bullet\mu_0 M_{sat}$. Under low-field approximation, the force on a spherical ferromagnetic particle is given by equation (2):

$$F = \frac{V\Delta\chi}{2\mu_0}\nabla(B_0)^2 = \frac{3VB_0}{\mu_0}.\frac{dB_0}{dR} \qquad (2)$$

In a high external magnetic field that is above the saturation field for the magnetic material of the MNP, the force equation can be written as:

$$F = \frac{1}{2}\nabla(m_{sat}B_0) = \frac{V}{2}M_{sat}\frac{dB_0}{dR} \qquad (3)$$
