# Supplementary material for: Non-Temperature Induced Effects of Magnetized Iron Oxide Nanoparticles in Alternating Magnetic Field in Cancer Cells
Source: PLoS One. 2016 May 31;11(5):e0156294. doi: 10.1371/journal.pone.0156294 (PMC4887104; doi:10.1371/journal.pone.0156294)
Supplement: S4 Appendix — The heating of conductive MNP in variable magnetic field. (PDF) [file pone.0156294.s004.pdf]

S4 Appendix. Heating effects

Heating of conductive magnetic particles in the variable magnetic field is due to three different mechanisms, including hysteresis heating, eddy current or induction heating, and ferromagnetic resonance. Hyperthermia with SPION is typically achieved by the hysteresis heating, and the cyclic increase in the internal energy can be defined as:

$$\Delta U = -\mu_0 \oint M dH, \qquad (4)$$

where, $M(t) = \mathrm{Re}[\chi H_0 e^{-i\omega t}] = H_0(\chi' \cos\omega t + \chi'' \sin\omega t)$, $H(t)=H_0 \cos\omega t$, and the complex susceptibility is $\chi=\chi' - i\chi''$. In the strong saturating magnetic field B_0, the magnetization $M = M_{sat} = const$, so there is no hysteresis heating.

The eddy current heating can be estimated using the equation:

$$P = \frac{\pi^2 B^2 d^2 f^2}{6k\rho D}, \qquad (5)$$

where P (W/kg) is the deposited power, B is the magnetic field amplitude, d is the characteristic size of the object, $f= \omega/2\pi$ is the frequency, ρ and D are the resistivity and the density of the material, respectively, and k is an empirical geometry factor between 1 and 2. For SPION with $d=50$ *nm*, $D = 5000\ kg/m^3$, $B = (G\bullet\Delta\chi)\sim 0.01T$, $\rho\approx 0.1\Omega m$, and f=5.4 kHz, the power deposition is $P\sim 10^{-14}\ W/kg$, which should not produce any measurable sample heating. Inductive heating of the cell sample by eddy currents in the conductive growth medium can be estimated as $P\sim 5\bullet 10^{-4}\ W/kg$ ($d=1\ cm$, $D = 1000$ *kg/m3*, $\rho \approx 0.62\ \Omega m$) and is negligible, as confirmed by our experimental results with control unlabeled cells.

Finally, the ferromagnetic resonance frequency is typically in the range of ~ $10^8 - 10^{10}$ Hz, and this mechanism does not contribute to the heating produced by AMF applied at audio frequencies.
